# A Compendium of Information on the Lysosome

**DOI:** 10.3389/fcell.2021.798262

**Published:** 2021-12-15

**Authors:** Nadia Bouhamdani, Dominique Comeau, Sandra Turcotte

**Affiliations:** ^1^ Department of Chemistry and Biochemistry, Université de Moncton, Moncton, NB, Canada; ^2^ Dr. Georges-L. Dumont University Hospital Centre, Clinical Research Sector, Vitalité Health Network, Moncton, NB, Canada; ^3^ Atlantic Cancer Research Institute, Moncton, NB, Canada

**Keywords:** lysosome, autophagy, endocytosis, reformation, cancer, BORC, mTOR, TFEB

## Abstract

For a long time, lysosomes were considered as mere waste bags for cellular constituents. Thankfully, studies carried out in the past 15 years were brimming with elegant and crucial breakthroughs in lysosome research, uncovering their complex roles as nutrient sensors and characterizing them as crucial multifaceted signaling organelles. This review presents the scientific knowledge on lysosome physiology and functions, starting with their discovery and reviewing up to date ground-breaking discoveries highlighting their heterogeneous functions as well as pending questions that remain to be answered. We also review the roles of lysosomes in anti-cancer drug resistance and how they undergo a series of molecular and functional changes during malignant transformation which lead to tumor aggression, angiogenesis, and metastases. Finally, we discuss the strategy of targeting lysosomes in cancer which could lead to the development of new and effective targeted therapies.

## 1 Introduction

In the past decade, the scientific community has shown an exponentially growing interest for lysosome research. Ground-breaking studies have highlighted the stunning intricacy of lysosome biology and functions, deviating from their initial and rather elemental role as garbage disposals for cellular components. Fortunately, we now know that lysosomal functions are as complex as they are fascinating, ranging from the degradation of extracellular and intracellular components to the crucial nutrient sensing ensuring metabolic and cellular homeostasis. Deciphering and understanding all the available scientific literature dealing with the fields of lysosome and autophagy research can quickly become overwhelmingly complex. Accordingly, the main objective of this review is to facilitate the assimilation of the scientific knowledge known to date on lysosomes such as their physiology and their newly discovered cellular functions. This review will outline the basics, starting with the early historical background of lysosome discovery, their physiology and biogenesis as well as their known roles and functions. In addition, recent scientific discoveries are discussed here as well as lysosomal implications in disease development and progression, which will be propitious for new researchers, students, and specialists in the field. Finally, the latest research focusing on targeting lysosomes in the context of cancer and how this strategy could become a promising approach for the development of new and effective targeted therapies is also reviewed.

## 2 Discovery

**FIGURE 1:**
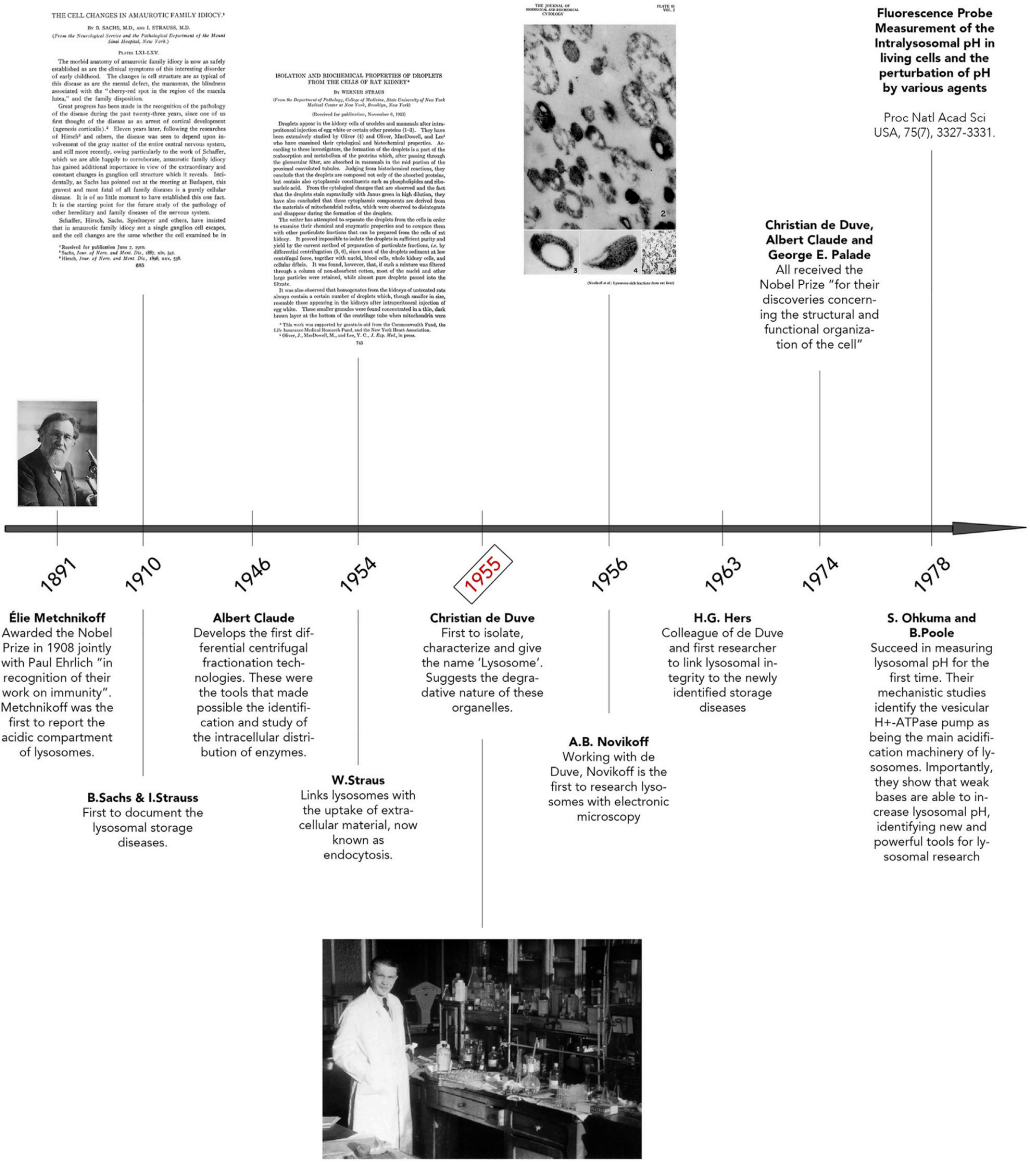
Discovery of Lysosomes. Timeline illustrating key breakthroughs in research that led to lysosomal identification and discovery. Important pioneers of lysosome research are highlighted here, including Élie Metchnikoff, Albert Claude, and Christian de Duve. The photo of Élie Metchnikoff is reproduced with permission from Mary Evans Picture Library. The title page of “The Cell Changes in Amaurotic Family Idiocy” was reproduced with permission from The Rockefeller Institute for Medical Research New York (^©^ Copyright 1910 Sachs B, Strauss I. Originally published in *J Exp Med*. 1910; 12(5):685–695. https://doi.org/10.1084/jem.12.5.685). The title page of “Isolation and biochemical properties of droplets from the cells of rat kidney” was made freely available under the Creative Commons Attribution 4.0 International Public License (Strauss w. Originally published in *J Biol Chem*. 1954; 207(2):745–755. https://doi.org/10.1016/S0021-9258(18)65693-5). The photo of Christian de Duve, entitled “Christian de Duve in his laboratory”, was reproduced freely from the Digital Commons at Rockefeller University. Plate 61 seen in “Electron Microscopy of Lysosome-rich Fractions from Rat Liver” was reproduced with permission from The Rockefeller Institute for Medical Research (^©^ Copyright 1956 Novikoff AB, Beaufay H and deDuve C. Originally published in *The Journal of biophysical and biochemical cytology*, *2*(4 Suppl), 179–184.

The early historical background of lysosome discovery began in the late 19th century with microscopic observations by Élie Metchnikoff (Figure 1) (Metchnikoff, 1893). Regarded by scientists as the father of natural immunity, Metchnikoff initially reported the uptake of foreign particles into cells and their subsequent digestion (Maxfield, 2016; Underhill et al., 2016). By using pieces of litmus, Metchnikoff observed the chemical change from blue to red, implying an acidic environment surrounding the ingested particles, thus documenting the acidity of lysosomes. Afterwards, B. Sachs and I. Strauss unknowingly documented phenotypic variances of lysosomes, describing unusual cellular accumulations of material in patient tissues. In reality, they were observing characteristics of what we now refer to as Lysosomal Storage Diseases (LSD) (Figure 1) (Sachs and Strauss, 1910). In 1946, Albert Claude developed the first differential centrifugation protocol to study the intracellular distribution of enzymes. (Figure 1) (Bainton, 1981). In fact, Christian de Duve’s pioneering lysosome discovery was made possible with Claude’s innovative techniques. It is also of importance to highlight the works of Werner Straus, a researcher who isolated lipid “droplets” rich in acid phosphatase (a ubiquitous lysosomal enzyme) from rat kidneys after intraperitoneal injection of egg white (Straus, 1954, 1956). Straus’ isolation of acid phosphatase-enriched droplets occurred independently of and at a similar time as Christian de Duve’s isolation and identification of lysosomes. Indeed, de Duve accidentally identified the lysosome when trying to purify a liver phosphatase specific for glucose-6-phosphate (Figure 1) (Claude, 1946a; 1946b). By attempting to clarify the role of insulin on the liver, De Duve’s group realized the activity of the enzyme acid phosphatase was latent and only membrane-disrupting treatments or repeat freeze-thawing of the samples were methods that caused a significant increase in the enzyme’s activity (Bowers, 1998; de Duve, 2005; De Duve et al., 1955; Sabatini and Adesnik, 2013). Thus, de Duve concluded that the enzyme was sequestered within “membrane sacs” (Berthet et al., 1951; Berthet and De Duve, 1951). By optimizing Claude’s fractionation protocol, de Duve succeeded in purifying the lysosome organelle. Another important step forward was Alex Novikoff’s work who carried out the first electron microscopic studies of the newly isolated organelle (Figure 1) (Novikoff et al., 1956).

Following the lysosome discovery, soaring interests in B. Strauss and I. Sachs’ previous studies re-surfaced. Hypotheses that lysosome defects could explain their previously documented accumulations of material in various patient tissues were emerging. This clear correlation was shown for the first time by Hers in 1963, when the defect of a glycogen storage disease was linked to a deficiency in the lysosomal enzyme, acid maltase, also known as *Pompe’s Disease* (Hers, 1963; Lejeune et al., 1963). In 1974, Christian de Duve, Albert Claude and George Palade all received the Nobel Prize “*for their discoveries concerning the structural and functional organization of the cell*” (Bowers, 1998; Sabatini and Adesnik, 2013). Finally, in 1978, Ohkuma and Poole elucidated the requirement of ATP for lysosomal acidification and that weak bases could increase the pH of these organelles (Ohkuma and Poole, 1978). This excellent study led to the identification of the vacuolar H+ ATPase pump (V-ATPase), whose main role consists in lysosome acidification. Following this period, from the 1980s until the early 2000s, extraordinary works were accomplished related to identifying the machinery of membrane traffic on the biosynthetic and endocytic routes to lysosomes (Kielian et al., 1986; Chavrier et al., 1990; Schu et al., 1993; Ullrich et al., 1994; Méresse et al., 1995; Horiuchi et al., 1997; Christoforidis et al., 1999; Lewis et al., 2000). Clear examples amongst many are the characterization of the vacuolar protein sorting (VPS) genes in yeast, mammalian orthologs of which were subsequently shown to make up much of the core machinery for delivery of cargo to and from lysosomes, the identification of the ESCRT proteins, crucial for multivesicular body (MVB) formation and the identification of the small Rab GTPases and their regulators and effectors necessary for the endocytic pathway to lysosomes (Katzmann et al., 2001). Excitingly, with a growing interest in autophagic processes, the past 20 years has seen a re-ignited enthusiasm for the characterization and study of lysosomes.

## 3 Degradation - The Crosstalk Between Endocytosis and Autophagy

**FIGURE 2:**
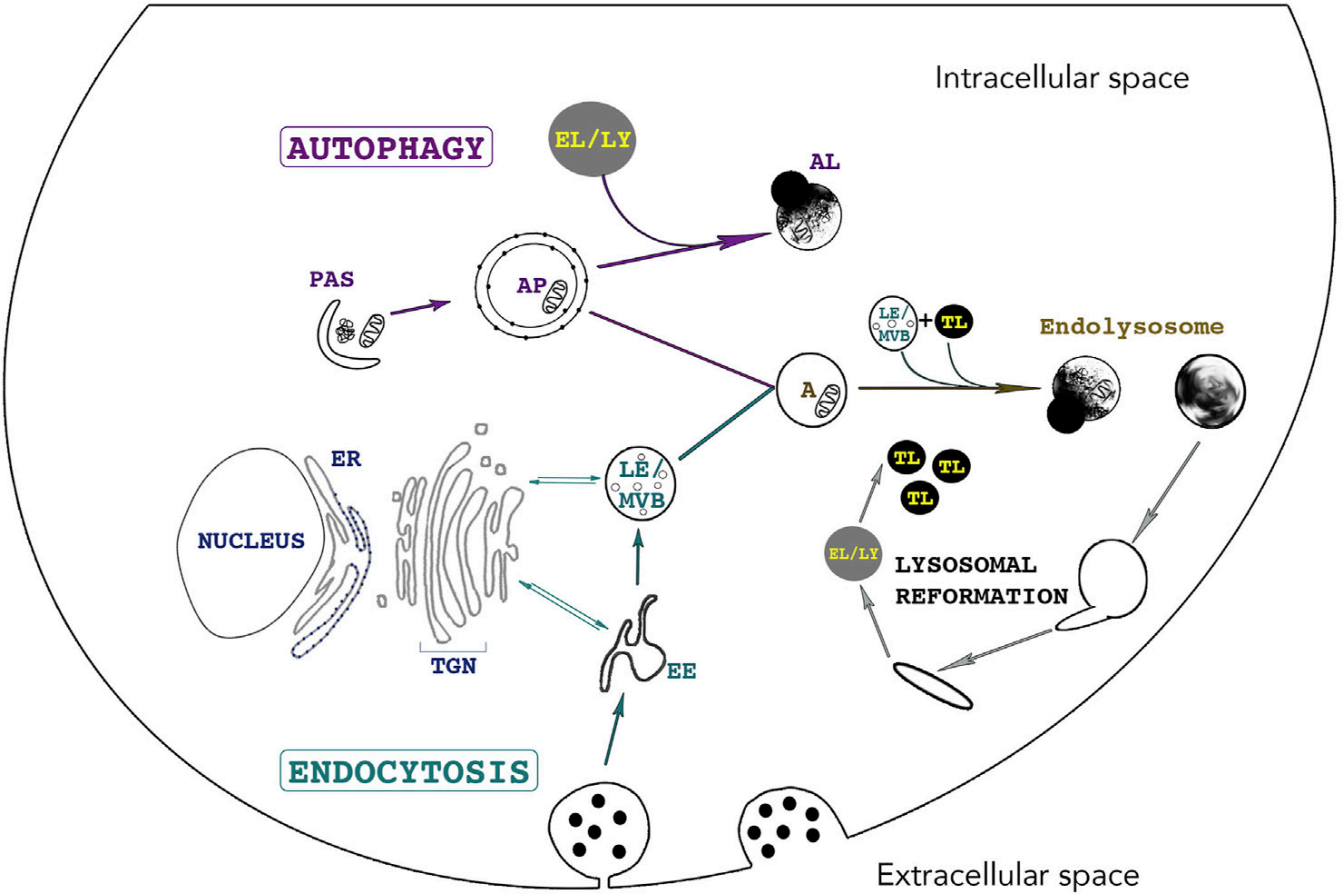
The lysosome - an indispensable shared organelle for autophagy and endocytosis. Autophagy and Endocytosis, two intimately linked cellular pathways leading to lysosomal degradation of intracytoplasmic and extracellular material, respectively. Autophagy is initiated by the formation of the phagophore also called *Pre-Autophagosomal-Structure* (PAS) derived from various membranes sources such as the endoplasmic reticulum, the golgi and the plasma membrane. PAS elongation and closure leads to bulk sequestration of intracytoplasmic material in the newly formed double membraned Autophagosome (AP). The AP can either directly fuse with the lysosome (LY) for degradation or can fuse with an endosome (Early Endosome (EE) /Late Endosome (LE)), creating an Amphisome (A). Endocytosis starts with the invagination of extracellular material and the budding of an endocytic vesicle from the plasma membrane. Endosome maturation is essential for lysosomal integrity. EE mature into LE and Multivesicular bodies (MVBs) by continuously maintaining vesicle trafficking with the Trans-Golgi Network (TGN). This trafficking is crucial for lysosomal integrity as it brings newly synthesized lysosomal enzymes and hydrolases to lysosomes to maintain an acidic pH and efficient degradative functions. Endosomes can directly fuse with lysosomes or with an AP. The fusion of LE and terminal storage lysosomes (TL) creates the principal intracellular site of acid hydrolase activity (EL) (Bright et al., 2016) Tubules emanate from EL (or AL) and lead to the reformation of terminal storage lysosomes, which are hydrolase-inactive and appear as dense bodies in electron microscopy.

Endocytosis and autophagy maintain cellular homeostasis by delivering extracellular and intracellular materials to lysosomes, respectively (Figure 2 **)**. Endocytosis is responsible for internalizing and processing external cues (*e.g.* fluid, solutes, plasma membrane components and particles) (Huotari and Helenius, 2011). Subtypes of endocytic routes differ by protein complexes including clathrin- and/or dynamin-dependent and -independent pathways. These subtypes are not the focus of this review and are described in-depth elsewhere (Steinman et al., 1983; Maxfield and Yamashiro, 1987; Doherty and McMahon, 2009; Kumari et al., 2010; Huotari and Helenius, 2011; Lamb et al., 2013; Ferreira and Boucrot, 2018; Kaksonen and Roux, 2018; Wallroth and Haucke, 2018). In brief, endocytosis begins with the budding of an endocytic vesicle from the plasma membrane and fuses with an early endosome (EE). This internalized material will meet different fates by trafficking to various compartments: to late endosomes (LE), multivesicular bodies (MVB) and to lysosomes for degradation while proteins that need recycling will travel back to the plasma membrane through either a fast route involving EEs or a slower pathway involving recycling endosomes (RE) (Kumari et al., 2010; Bento et al., 2016). Lysosomal proteins are synthesized in the ER, processed in the Golgi complex and enter the Trans Golgi Network (TGN). Trafficking between endosomes and the TGN is imperative for the maintenance of lysosomal fitness as it delivers enzymes and active V-ATPase pumps to lysosomes via the endocytic route and removes endosomal components during endosome maturation (Huotari and Helenius, 2011; Dutta and Donaldson, 2012). Soluble lysosomal enzymes are modified in the Golgi complex with residues of mannose-6-phosphate (M6P). Following their entry in the TGN, these enzymes bind a M6P receptor (MPR) after which a heterotetrameric adaptor protein complex (AP)-1 and the Golgi-localized, γ -ear-containing, Arf-binding (GGA) protein family, recognizes specific motifs in the cytosolic tail of the MPRs and recruits clathrin. This step permits MPRs to enter clathrin-coated vesicles and travel to endosomes (Bonifacino and Traub, 2003; Ghosh and Kornfeld, 2004a, 2004b).

Several subtypes of autophagy are characterized, namely Chaperone-Mediated Autophagy (CMA), macroautophagy and microautophagy amongst others (Cuervo, 2004; Kaushik and Cuervo, 2012). Macroautophagy (hereafter referred to as autophagy) is the most studied subtype of autophagy and is characterized by the sequestration of cytoplasmic cargoes in a double-membraned vesicle called the autophagosome (AP). The autophagy-related (ATG) proteins assemble at a site called the preautophagosomal structure or phagophore assembly site and together with specific SNARE (soluble *N-*ethylmaleimide-sensitive factor attachment protein receptors) proteins, they mediate the formation of a phagophore by orchestrating fusion of ER-, Golgi-, and plasma membrane -derived membranes (Figure 2) (Moreau et al., 2011; Nair et al., 2011; Puri et al., 2013; Bento et al., 2016; Pavel and Rubinsztein, 2017). The elongation and closure of the phagophore sequesters cytoplasmic components and forms the AP. APs fuse with endosomes to form amphisomes and then with lysosomes, creating autolysosomes (AL). Crosstalk between autophagy and endocytosis occurs at many levels as several ATG and endocytic proteins have indispensable roles in both pathways. For example, interactions between endocytic vesicles, ATG proteins and REs occur at early stages of AP biogenesis. Indeed, two essential ATG proteins, ATG16L1 and plasma-membrane-associated ATG9, bind distinct pools of clathrin-coated endocytic vesicles before associating with Rab11-positive REs and EEA1-positive EEs, respectively (Moreau et al., 2011; Longatti et al., 2012; Puri et al., 2013). The fusion of ATG9- and ATG16L1-positive membranes is mediated by the SNARE protein VAMP3, localized at REs. In fact, its knockdown results in the accumulation of ATG9 in EEs and results in a decrease in AP formation. Furthermore, endocytosis assures an efficient autophagic flux as endosome maturation enables lysosomes to acquire newly synthesized hydrolases, V-ATPases, transporters and permeases (Lamb et al., 2013).

## 4 Lysosome Physiology

Today, lysosomes are known to be a highly heterogeneous collection of organelles, differing by their localization and acidity, their functions as well as by the hybrid organelles they form through fusion/fission and “kiss and run” events (Saffi and Botelho, 2019). Several different terms are now used to differentiate these lysosomal compartments (*e.g.* endolysosomes, phagolysosomes, autolysosomes; or even to distinguish acidic (endolysosomes) vs. non-acidic (terminal lysosomes) lysosomes) (Bright et al., 2016; de Araujo et al., 2020). For comprehensibility, terms used in this review will be clearly defined. Endocytic compartments such as EE mature to become LE which are only moderately acidic. The fusion of LE and terminal storage lysosomes creates a hybrid organelle named the endolysosomes, the principal intracellular site of acid hydrolase activity (Bright et al., 2016). Tubules emanate from EL (or AL) and lead to the reformation of terminal storage lysosomes, which are hydrolase-inactive and appear as dense bodies in electron microscopy (Tjelle et al., 1996). Thus, homeostasis between the formation of terminal storage lysosomes and the number of EL is crucial to maintain adequate cellular degradative capacity. The term ‘lysosome’ will thus be used broadly unless precisely specified.

### 4.1 Overview

**FIGURE 3:**
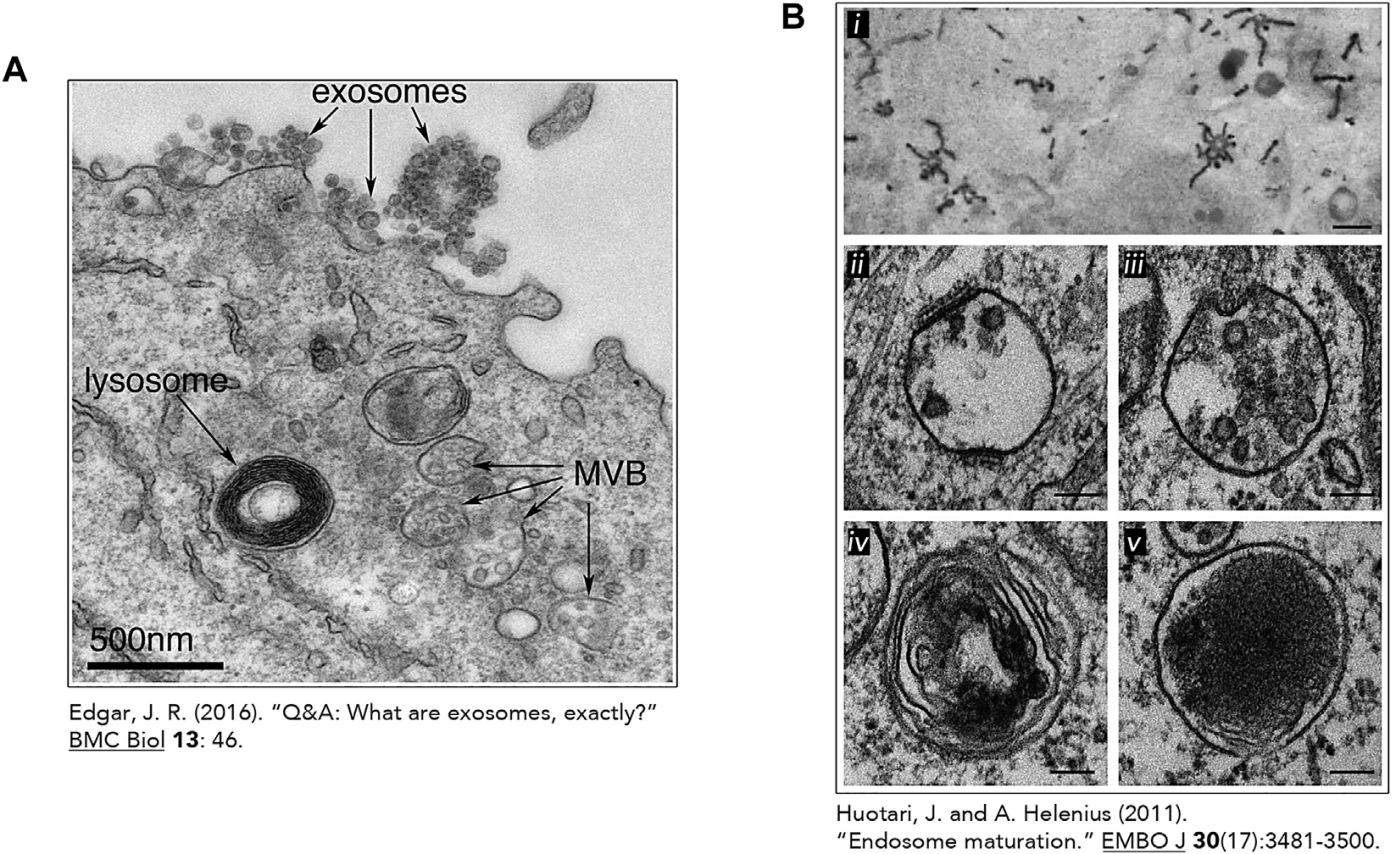
The heterogeneous nature of the lysosome. Transmission electron micrographs of lysosomes and other related organelles (Huotari and Helenius, 2011; Edgar, 2016). **(A)** Lysosome identified by an arrow in an Epstein-Barr virus-transformed B cell displaying an increase exosome release. This image was made freely available under the Creative Commons Attribution 4.0 International Public License (Edgar JR. Originally published in *BMC Biology*. 2016; 14:46. DOI 10.1186/s12915-016-0268-z). **(B)** Heterogeneous morphologies of endosomes and lysosomes. **(i)** Early Endosomes (EE) containing HRP-conjugated Transferrin (Tf) (500 nm) **(ii)** Early Endosomes containing intraluminal vesicles (ILV) and clathrin lattices (100 nm) **(iii)** Late Endosome and its associated ILVs. (100 nm) **(iv)** Endolysosome (by fusion of a lysosome and endosome) (100 nm). **(v)** Micrograph of lysosomes with electron dense lumen. (100 nm). **(B) (i)** was reproduced with permission from The Rockefeller University Press (^©^ Copyright 1992 Tooze J and Hollinshead M. Originally published in *The Journal of cell biology*, *118*(4), 813–830. https://doi.org/10.1083/jcb.118.4.813). Figure 3B (*ii-v*) was reproduces with permission from EMBO (Huotari, J., and Helenius, A. (2011). Endosome maturation. *The EMBO journal*, *30*(17), 3481–3500. https://doi.org/10.1038/emboj.2011.286).

In nutrient-rich conditions, these digestive compartments are heterogeneous in size, morphology, and distribution. Lysosome shape can vary from spherical to tubular and their number differ depending on conditions and cell types (Figure 3) (Appelqvist et al., 2013). In fact, when adopting a vesicular shape, lysosomes have a diameter ranging from 0.5 μ m to >1 m μ . Interestingly, tubular lysosomes can reach lengths of >15 m μ under certain conditions, as seen in phagocytes (Hipolito et al., 2018). Several hundred lysosomes can be counted in a single cell; however, upon nutrient deprivation and when autophagy is induced, lysosome number decreases dramatically to <50 per cell and increase in size because of lysosomal consumption and membrane fusion, respectively (Mellman, 1989; Bandyopadhyay et al., 2014). Lysosomes contain over 60 types of hydrolytic enzymes (*e.g.* peptidases, phosphatases, nucleases, sulfatases, lipases, proteases and glycosidases) with different target substrates assuring the degradation of all types of macromolecules (Saftig and Klumperman, 2009; Schwake et al., 2013). Degradation products are transported out of lysosomes by specific exporters in the limiting membrane or via vesicular membrane trafficking; these products serve in the maintenance of energy homeostasis or are reutilized in biosynthetic pathways (Ruivo et al., 2009). The best characterized lysosomal hydrolases are the family of cathepsin proteases and are classified in three distinct groups based on the amino acid (AA) found in the active site: serine (A and G), cysteine (B, C, F, H, K, L, O, S, V, W, and X) and aspartic cathepsins (D and E) (Appelqvist et al., 2013). The latter and some cysteine cathepsins (B, C, H, and L) are ubiquitous and are amongst the most abundant proteases (Rossi et al., 2004). The acidic pH of lysosomes (pH 4.5–5.0) is optimal for the activity of these hydrolases (Coffey and De Duve, 1968; Ohkuma and Poole, 1978). This is assured by the V-ATPase, a transmembrane multimeric protein complex that uses energy from ATP hydrolysis to pump protons from the cytosol into the lysosomal lumen (Ohkuma et al., 1982; Mindell, 2012). Lysosomes are mainly made up of glycerophospholipids and sphingolipids and contain high contents of bis(monoacylglycero)phosphate (BMP). The latter ranges from 4 to 17% and up to 70% of the total lysosomal lipid content in the external limiting membrane and the intraluminal vesicles, respectively. The single 7–10 nm external phospholipid-bilayer comprises high carbohydrate content due to heavily glycosylated lysosomal membrane proteins forming a glycocalyx (Saftig et al., 2010). The glycocalyx maintains lysosomal integrity as it protects the membrane from lytic enzymes found in the lysosome.

### 4.2 Lysosomal Membrane Proteins

Lysosomal membrane proteins are synthesized in the ER and make their way to the lysosome through several clathrin dependent and independent pathways. Following synthesis in the ER and glycosylation, they enter the TGN and can either follow the secretory route to the plasma membrane for reinternalization by endocytic processes or can be directly transported to lysosomes through endosomal compartments (Carlsson and Fukuda, 1992; Harter and Mellman, 1992). To date, more than 100 lysosomal membrane proteins have been identified through proteomics (Lübke et al., 2009; Schröder et al., 2010). The most abundant are the type-1 transmembrane proteins harboring more than 10 glycosylation sites, the lysosome-associated membrane protein (LAMP)-1 and -2, lysosomal integral membrane protein (LIMP)-2, and CD63 (LIMP1) (Eskelinen et al., 2003). These proteins have been recognized for their important roles in transport processes across the lysosomal membrane, for instance, knockout of LAMP proteins result in a significant accumulation of lysosomal cholesterol (Eskelinen et al., 2004; Schneede et al., 2011). The transmembrane segments of these proteins are generally implicated in transport events across the membrane while the short cytosolic part of lysosomal membrane proteins mediates interactions with cytosolic proteins and/or proteins present on other organelles (Schwake et al., 2013). LAMP proteins were also shown to stabilize a polypeptide translocation machinery (TAPL), required for the transport of cytosolic peptides assuring their lysosomal degradation (Demirel et al., 2012). Moreover, a specialized autophagic pathway, chaperone-mediated autophagy (CMA), which assures the lysosomal degradation of approximately 30% of all cytosolic soluble proteins containing a KFERQ sorting motif, is executed by one of the three isoforms of LAMP-2, LAMP-2A (Kaushik and Cuervo, 2012). Other known roles of LAMP proteins include lysosome motility, lysosomal exocytosis as well as lysosome fusion with the plasma membrane (Huynh et al., 2007; Yogalingam et al., 2008).

**FIGURE 4:**
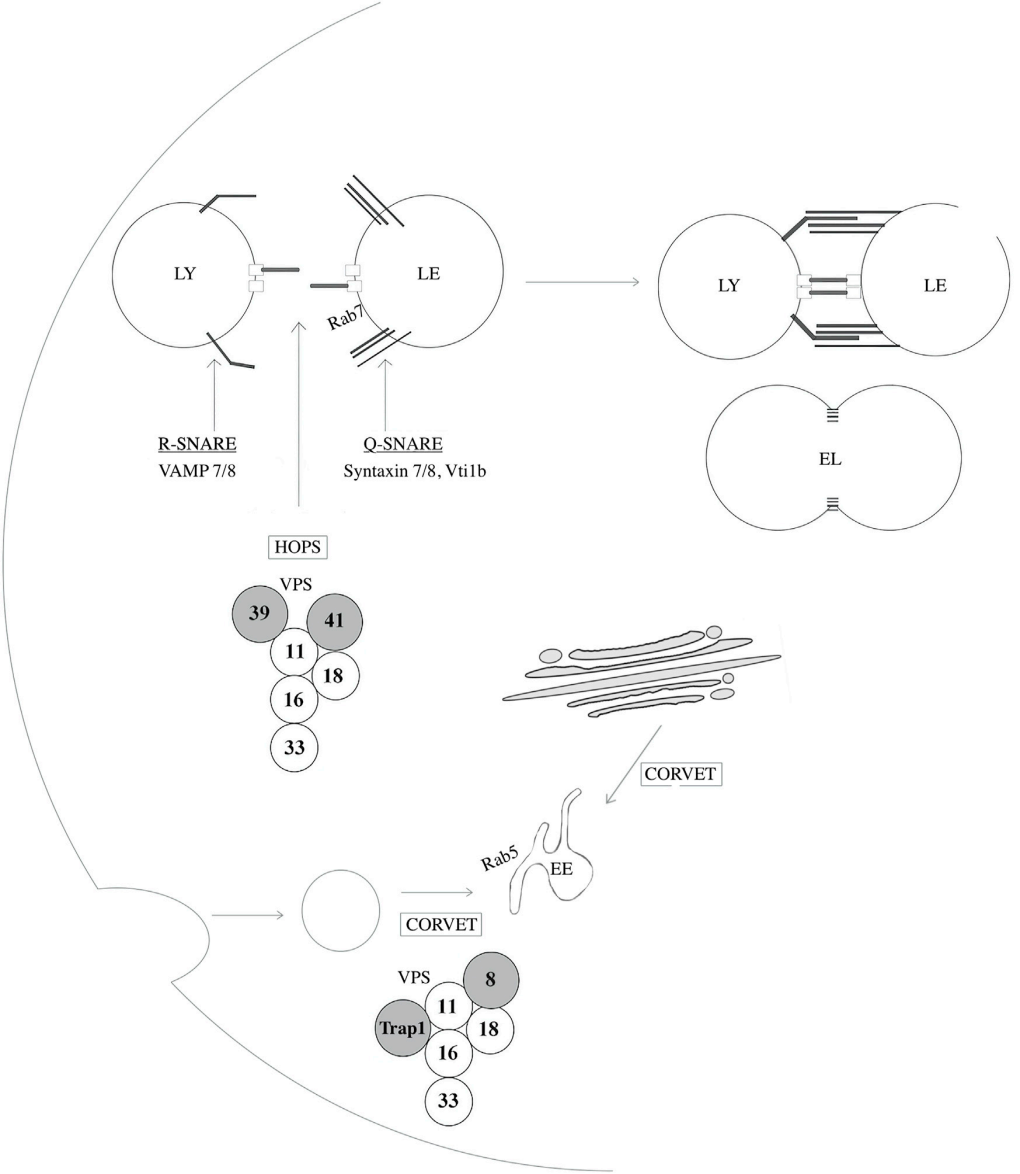
Proteins implicated in lysosome-endosome fusion events. SNAp REceptor (SNARE) proteins regulate fusion events between lysosomes and other organelles such as late-endosomes, autophagosomes, and amphisomes. VAMP7 and VAMP8 are R-SNAREs, a type of SNARE proteins that provides an arginine (R) residue in the assembly of the SNARE complex and are located at the lysosomal membrane. Syntaxin 7, Syntaxin 8 and VTI1B are Q-SNARES, providing a glutamine (Q) residue in the assembly of the SNARE complex, and localize to late endosomes (LE). The homotypic fusion and vacuole protein sorting (HOPS) tethering complex assures heterotypic fusion between endosomes and lysosomes (LY) by first recognizing and interacting with Rab7. Similarly, the class C core vacuole/endosome tethering (CORVET) mediates homotypic fusion of Rab5-positive early endosomes (EE). These two tethering complexes share core members, VPS11, 16, 18, and 33A/B while HOPS and CORVET uniquely contains VPS39 and VPS41 and VPS8 and TRAP-1, respectively. EL: Endolysosome.

Other lysosomal membrane proteins include various transporters and exchangers such as cystinosin, sugar channels including spindling (SPIN), AA transporters including SLC38A9, LYAAT-1, LAAT-1 and SNAT7, ion channels including mucolipin 1 (MCOLN1, also known as TRPML1), assuring the regulation of calcium levels as well as stores of AA, sugars and ions (Cu^+^, Fe^+^), the well-characterized multi-subunit vacuolar type H+ATPase (v-ATPase), responsible for the transport of protons into the lysosomal lumen, and some newly identified proteins such as DIRC2, which is suggested to be involved in electrogenic transport across the lysosomal membrane (Lawrence and Zoncu, 2019; Schwake et al., 2013). In addition to lysosomal transmembrane proteins, the lysosomal membrane accommodates several other proteins such as tethering factors and SNARE proteins which is crucial for fission and fusion events (Bröcker et al., 2010; Hong and Lev, 2014). Lysosomal fusion events with target organelles are mediated by the assembly of four SNARE proteins; a parallel four-helix bundle that forms a SNAREpin (also known as a *trans*-SNARE complex). Fusion with late endosomes is mediated by the assembly between an R-SNARE (VAMP7 or VAMP8) located at the lysosomal membrane, and three Q-SNAREs (syntaxin 7, syntaxin 8, and VTI1B) located on LEs, or similar constituents on autophagosomes (SNAP27, Syntaxin 17). (Figure 4 **)** Membrane fusion occurs following a calcium-dependent conformational change of the SNAREpin which is later disassembled by the AAA ATPase N-ethylmaleimide-sensitive factor (Luzio et al., 2007). Lastly, a recently identified complex, the multi-subunit protein complex BLOC-1-related complex (BORC), was also shown to localize to the lysosomal membrane and mediate movement through physical interaction with kinesin-5 via ARL8. This protein complex will be further described in the sections below (Pu et al., 2015).

### 4.3 pH, Acidification, Ion Flux and Transporters

The acidity and degradative capacity of lysosomes stem from the complex establishment of an acidic endosomal lumen during endosome maturation. This process requires a dynamic collection of channels, transporters, and exchangers at the limiting membrane of endosome compartments. Alterations in lysosomal ion channels and ion flux have important repercussions on lysosome motility, membrane trafficking and repair, nutrient sensing, organelle membrane contact, lysosome biogenesis and overall homeostasis (Li et al., 2019). While the v-ATPase assures an electrogenic proton translocation in endolysosomes, a flux of ions other than protons are needed as a compensatory flow of charge. Without this flux of ions, the transmembrane voltage generated by the v-ATPase would simply form a self-limiting electrochemical gradient and consequently impede luminal acidification. Along the endocytic pathway, there are continuous changes in ionic composition, starting at the nascent endocytic vesicle budding from the plasma membrane to early endosome and multi-vesicular body formation and finally at late endosomes and terminal storage lysosomes. Differing concentrations of ions have been measured in each endosomal compartment such as a significant decrease of sodium (Na^+^, 120 mM at the nascent endocytic vesicle to less than 20 mM in lysosomes) coupled with an increase in potassium (K^+^, 5 mM at the nascent endocytic vesicle to 60 mM in lysosomes). Two additional ions that seem to play crucial roles in endocytic processes and acidification are chloride (Cl^−^) and calcium (Ca^2+^). Interestingly, the concentration of these ions are higher in the extracellular fluid and decreases dramatically after vesicle formation and as measured in the early endosome (Cl^−^, 130 mM at the PM to 19 mM in the EE; Ca^2+^, 1 mM at the PM to ∼5 μ M in the EE). However, from EE to MVB formation and to endolysosomes where acidity is at its highest, it is believed that there is a steady increase in Cl^−^ and Ca^2+^ concentrations (Gerasimenko et al., 1998; Christensen et al., 2002; Weinert et al., 2010).

Specific transporters have in fact been linked to the regulation of endolysosomal function through pH regulation. The major active ion transporters within the endocytic pathway are 1) the electroneutral NHE sodium (potassium)/proton exchangers, 2) the ClC chloride anti-porters, 3) members of the transient receptor potential (TRP) superfamily of calcium channels such as the mucolipins (TRPMLs) and 4) members of the two-pore channels (TPCs), other known endolysosomal Ca^2+^ channels. The latter channels have been shown to modulate lysosomal pH, directly linking TPCs to endosome function (Morgan and Galione, 2007). Mechanistically, the function of the NHE sodium/potassium exchangers remains debatable; however, it is likely that they exchange Na^+^ or K^+^ for H^+^ in endosomal and Golgi compartments which influences pH and salt concentrations in endocytic compartments. Finally, several ClCs have been identified along the endocytic pathway (EE, RE, and at the lysosome) and their function has also been linked to endolysosomal pH (Mohammad-Panah et al., 2003; Hara-Chikuma et al., 2005; Graves et al., 2008). Identifying which transporters are responsible for generating a compensatory flux of ions to assure endosomal acidification has been difficult for many reasons such as the lack of proper probes or obvious discrepancies when tested *in vivo* (Mohammad-Panah et al., 2003; Hara-Chikuma et al., 2005; Kasper et al., 2005; Graves et al., 2008; Steinberg et al., 2010; Weinert et al., 2010; Wellhauser et al., 2010). More recently, TPCs (TPC1, TPC2) have been shown to be sodium selective and activated by a low-abundant phosphoinositide, PI(3,5)P_2_, linking phosphoinositides to pH regulation (Wang et al., 2012). To date, two potential mechanisms have been proposed to explain the modulation of endocytic functions through pH acidification by ion exchange activity; the continuous major alterations in luminal ion concentrations along the endocytic pathway, and/or a pivotal role for a specific ion flux that could be dependent on spatiotemporal factors (Scott and Gruenberg, 2011). Nevertheless, the activation, permeability, selectivity and biophysical properties of these channels remain controversial and necessitate further investigation.

### 4.4 Lysosome Size

Lysosomes respond to several different intra- and extracellular stimuli by continuously adjusting their position, motility, numbers and size (de Araujo et al., 2020). The newly identified effectors of lysosome size are the phosphoinositides, more specifically, the phosphatidylinositol-3-phosphate (PI3P) and phosphatidylinositol-3,5-biphosphate (PI(3,5)P_2_). These lipids regulate precise spatiotemporal regulation of membrane dynamics as well as membrane and vesicular trafficking by modulating the recruitment and activity of specific effector proteins (Phan et al., 2019). The first evidence of an implication of phosphoinositides in mediating lysosome size was highlighted when the treatments of cells with weak base compounds such as chloroquine and wortmannin resulted in the swelling of lysosome structures (Fernandez-Borja et al., 1999; Ohkuma and Poole, 1981). Wortmannin inhibits PI3K, which consequently inhibits the synthesis of PI(3)P, the product of PI3K, essential for autophagy initiation as well as for the integrity of early and late endocytic compartments. PI(3)P regulates several aspects of the endosomal pathway such as the promotion of early to late endosomal transition (Poteryaev et al., 2010). In mammalian cells, PI(3,5)P_2_ account for approximately 0.08% of total inositol phosphoinositides, which complicates its study, isolation and quantification. Despite its low abundance, a deregulation of PI(3,5)P_2_ levels has devastating effects on endolysosomal structures and causes a significant enlargement of early and late endolysosomal compartments (Rutherford et al., 2006). In fact, PI(3,5)P2 has been shown to maintain the functionality of endocytic compartments and calcium channels, to mediate fusion and fission events, and even to assure lysosome turnover by mediating lysosome reformation (Ho et al., 2012). Importantly, the lipid kinase PIKfyve is the only enzyme able to synthesize PI(3,5)P_2_ from PI(3)P and its activity is assured by a protein complex comprising the enzyme itself, a pentameric ArPIKfyve (VAC14) scaffold and the antagonistic 5-phosphatase Sac3 (Fig4) (PAS complex) (Ikonomov et al., 2018; Ikonomov et al., 2009; Lees et al., 2020). Notably, although Fig4/Sac3 has an enzymatic role opposing PI(3,5)P_2_ biosynthesis, it is crucial for the stability and activity of the PAS complex; hence, a deletion/inhibition of the enzyme leads to a reduction of the lipid rather than an augmentation (Botelho et al., 2008; Chow et al., 2007). Aside from its structural role in the complex and its lipid phosphatase activity, Fig4 is also a protein phosphatase targeting PIKfyve, priming the enzyme. This explains why catalytically active Fig4 is necessary for the activity of the complex (Duex et al., 2006a; Duex et al., 2006b; Lees et al., 2020; Strunk et al., 2020). Furthermore, PIKfyve inhibits its lipid kinase activity by autophosphorylation which in turn stimulates Fig4 activity. The dynamic cues and players regulating this complex remains an avid area of research, with many unresolved questions. For instance, it would be important to identify what signals are responsible for the switch from kinase to phosphatase activity and how this avoids inefficacious cycles of ATP hydrolysis. Surprisingly, it has been shown that decreased levels as well as an accumulation of PI(3,5)P_2_ both result in swelling of endolysosome structures, demonstrating the importance of a fine-tuned phosphoinositide turnover. PIKfyve inhibition also causes important defects in retrograde transport and impedes lysosome reformation, two processes requiring efficient fission events (Rutherford et al., 2006; Bissig et al., 2017; de Araujo et al., 2020). Consequently, it is thought that endolysosomal swelling occurring in response to PIKfyve inhibition is a result of decreased fission events and inadequate lysosomal reformation (Bissig et al., 2017). PI(3)P assures fusion events between autophagosomes and endosomes and/or lysosomes, whereas PI(3,5)P_2_ has been shown to regulate specific ion channels which assures adequate calcium concentrations and acidification of endolysosomal compartments for ideal degradative functions. In fact, a recent study by *Yordanov et al.* showed the ability of BORC to regulate PIKfyve activity and consequently affect PI(3,5)P_2_ levels and thus, endolysosomal size (Yordanov et al., 2019). Specifically, by inhibiting core effectors of BORC, Dyaskedin and Myrlysin, the group showed an increased activation of the AMP-activated protein kinase (AMPK) resulting in increased levels of PI(3,5)P_2_, indicative of an increased activation of the PIKfyve kinase. Increased levels of PI(3,5)P_2_ activated the calcium channel TRPML1 at endolysosome membranes, causing a release in Ca^2+^ which consequently activated the protein Calcineurin enabling the dephosphorylation and nuclear translocation of the transcription factor EB (TFEB). TFEB activation led to an initiation of autophagic processes and most importantly to an increase in lysosomal reformation and subsequent reduction in lysosome size.

## 5 Lysosomal Functions

### 5.1 Nutrient Sensing

**FIGURE 5:**
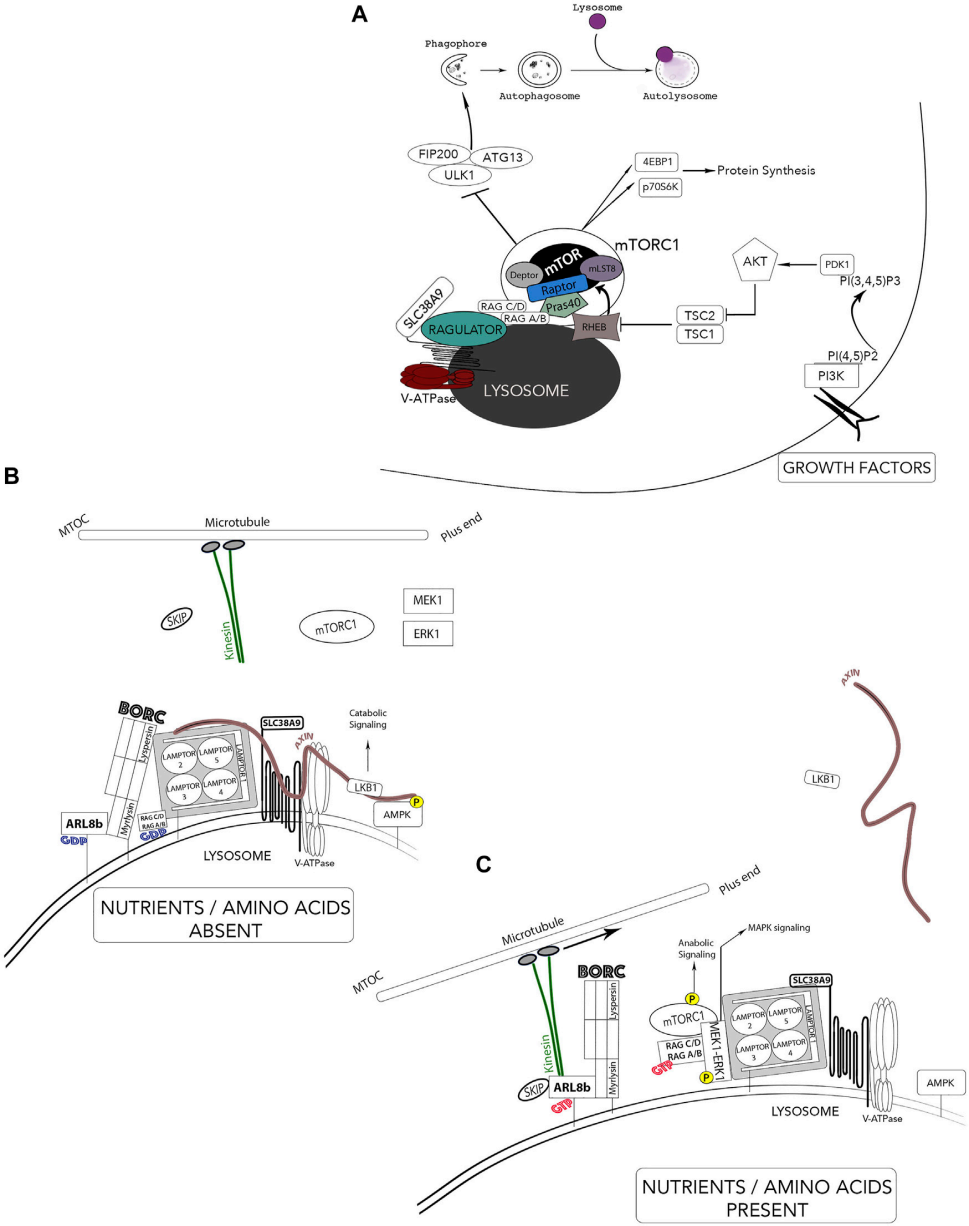
Lysosomes - Nutrient sensors via mTOR and autophagy regulation. The lysosomes receive information from the extracellular environment and generates a signaling response mediated by the activation of mTORC1. Two major independent signals regulate mTOR activity; growth-factor receptors and amino acids. **(A)** Growth factor receptors activate mTOR through PI3K and AKT pathways and leads to tuberous sclerosis complex (TSC1/2) and Rheb. mTOR is brought in proximity to its activator Rheb by both the Ragulator complex and Rag GTPases in response to elevated amino acid contents. Activated mTORC1 phosphorylates S6K and 4EBP1 and activates protein synthesis while inactivating mTORC1 by starvation inhibits anabolic processes and activates autophagy. **(B)** In the absence of nutrients and amino acids, Ragulator can activate catabolic pathways such as LKB1-AMPK signaling pathway through the scaffold Axin. The population of these lysosomes are thus located in the perinuclear area. **(C)** In the presence of nutrients and growth factors, the newly identified BORC complex and Axin dissociate from the Ragulator complex and leads to inactivation of catabolic processes (AMPK) and an association of BORC to the kinesin motors thus, increasing anterograde movement of lysosomes away for the microtubule organizing center (MTOC). *Adaptation of Colaco and Jaattela (2017).

A breakthrough in lysosomal research was achieved when a mechanistic co-regulation of cell growth and catabolism was established with the localization of the mammalian target of rapamycin (mTOR) at the lysosomal surface. Including mTOR, the proteins RAPTOR, mLST8, PRAS40 and DEPTOR comprises the mTOR complex 1 (mTORC1) (Figure 5). This complex assures anabolic processes such as protein, lipid, and nucleotide synthesis by mTOR-dependent phosphorylation of downstream effectors p70S6K and eukaryotic translation initiation factor 4E-binding protein (4EBP1) (Guertin and Sabatini, 2007; Laplante and Sabatini, 2012; Sancak et al., 2010). Growth-factor receptors activate mTOR through phosphatidyl-inositol-3-kinase (PI3K) and protein kinase B (PKB/Akt). Subsequent inhibition of the tuberous sclerosis complex (TSC1/2) leads to activation of the small GTPase Rheb which interacts and activates mTORC1 (Figure 5A). Regulating molecules control the metabolic status of the cell by using the lysosomal membrane as a signaling platform such as AMP-activated protein kinase (AMPK), ERK1/MAPK3 and MEK1 (Figures 5B,C) (Hamalisto and Jaattela, 2016; Medina et al., 2015; Zhang et al., 2013). Lysosomes also have the ability to sense amino acid availability through protein complexes that are localized at the lysosomal membrane. When amino acid levels are low, the appropriate signaling from the lysosomal membrane acts by shutting down anabolic pathways and activating catabolic routes such as the liver kinase B1 (LKB1)-AMPK signaling thus, initiating autophagy (Figure 5B) (Pu et al., 2017). Contrastingly, when amino acid levels are abundant, anabolic pathways are activated and mTORC1 suppresses the activity of the kinase ULK1 required for autophagosome biogenesis (Figure 5C).

Aside from growth factors, the main sensors of lysosomal luminal levels of AA (such as glutamine and arginine) are the V-ATPase and the lysosomal amino acid transporter/receptor SLC38A9 (Rebsamen et al., 2015; Rebsamen and Superti-Furga, 2016). The pentameric *Ragulator* complex interacts with both SLC38A9 and the V-ATPase for nutrient-sensing, more specifically, levels of lysosomal luminal AA activate Ragulator through an inside-out mechanism requiring SLC38A9 and the V-ATPase (Figure 5) (Korolchuk et al., 2011; Soulard and Hall, 2007; Tee et al., 2003a; Tee et al., 2003b; Zoncu et al., 2011). The Ragulator complex is composed of LAMTOR1 which anchors the complex to the lysosome and tightly holds together the heterodimers LAMTOR2/3 and LAMTOR 4/5 (Figure 5C) (Bar-Peled et al., 2012). Importantly, Ragulator acts as a guanine nucleotide exchange factor and mediates the activation and docking of Rag GTPases (RagA/B and RagC/D) to the lysosomal membrane, which form obligate heterodimers in which RagA or B pairs with RagC or D (Figure 5C) (Inoki et al., 2003; Sancak et al., 2008; Bar-Peled et al., 2012; Schweitzer et al., 2015). Specifically, LAMTOR2/3 binds RagA/B and RagC/D which enables RagA/B to be GTP-bound; hence, recruiting mTORC1 to the complex for its activation. In nutrient-rich conditions, LAMTOR2/3 also binds MEK1, enabling the activation of ERK1. Ragulator also interacts with Axin, a scaffold protein for the LKB1-AMPK complex on the lysosomal surface. These interacting protein complexes reveal a clear role for the lysosome as a signaling hub. Thus, mTORC1 is brought in proximity to its activator Rheb by both the Ragulator complex and the Rag GTPases and enhances the association of mTORC1 with both late endosomal and lysosomal compartments (Kim et al., 2008; Sancak et al., 2008; Sancak et al., 2010). Inhibiting lysosomal functions cause the release of mTORC1 from the lysosome surface and the inactivation of its catalytic activity. In addition to its role as an activation platform for metabolic signaling, Ragulator now emerges as the protein complex responsible for lysosomal positioning (Colaco and Jaattela, 2017). Lysosomes can be divided in two distinct populations: a juxtanuclear pool, located close to the microtubule organizing center (MTOC) and a smaller peripheral population close to the plasma membrane. The latter is responsible for various cell type-specific functions (cell adhesion, cell motility, tumor invasion, cell death, metabolic signaling, cytosolic and extracellular pH, plasma membrane repair and bone resorption) while the juxtanuclear lysosomal pool assures “housekeeping” degradative functions (Kallunki et al., 2013). Rubinsztein and colleagues, the first to link lysosomal movements to autophagy modulation, showed that lysosomes respond to amino acids and growth factors by moving to the plasma membrane while starvation conditions resulted in their perinuclear localization (Korolchuk et al., 2011; Korolchuk and Rubinsztein, 2011).

Recently, a lysosomal associated hetero-octameric protein complex named BLOC-1 related complex (BORC) was identified. BORC is comprised of BLOC1S1/BLOS1/BORCS1 (biogenesis of lysosomal organelles complex 1 subunit 1), BLOC1S2/BLOS2/BORCS2 (biogenesis of lysosomal organelles complex 1 subunit 2), SNAPIN/BORCS3 (SNAP associated protein), KXD1/BORCS4 (KxDL motif containing 1), BORCS5/myrlysin/LOH12CR1 (BLOC-1 related complex subuunit 5), BORCS6/lypersin/C17orf59 (BLOC-1 related complex subunit 6), BORCS7/diaskedin/C10orf32 (BLOC-1 related complex subunit 7) and BORCS8/MEF2BNB (BLOC-1 related complex subunit 8). Anterograde (centrifugal) and retrograde (centripetal) lysosomal movements along the microtubule tracks are mediated by plus end-directed kinesin motors and minus end-directed dynein motors, respectively. BORC links lysosomes to kinesin motors by activating the Arf-like GTPase Arl8 (Figures 5B,C) (Pu et al., 2017; Pu et al., 2015). Functionally, the active form of Arl8b (bound to GTP) localizes largely to the lysosome, where it recruits the kinesin-interacting linker protein SKIP, which in turn enables the downstream interaction of Kinesin 1 to lysosomes (Hofmann and Munro, 2006; Rosa-Ferreira and Munro, 2011). Thus, the BORC/arl8b/SKIP complex recruited to Kinesin promotes lysosomal transport toward the cell periphery (Farías et al., 2017; Pu et al., 2016; Pu et al., 2015). Two elegant studies elucidate how lysosome positioning changes in response to growth factor signaling and nutrient availability. Pu *et al.* and Filipek *et al.* show that nutrient availability and growth factor signaling increase the anterograde movement of lysosomes through a weakened BORC and Ragulator interaction, thus permitting the recruitment of Arl8/SKIP to the lysosomal surface. This enables the tethering of lysosomes to the kinesin motors and subsequent anabolic signaling (Figure 5) (Filipek et al., 2017; Pu et al., 2017). The elucidation of the intercommunication between these protein complexes at the lysosomal surface helped enormously in understanding the complexity of lysosome functions. However, some mechanistic questions remain unanswered such as which LAMTOR subunit interacts with the scaffold Axin.

The regulation of lysosome positioning by BORC has also led to the discovery of its role in the maintenance of autophagic flux by promoting both encounter and fusion of lysosomes with autophagosomes (Jia et al., 2017). Knockout of BORC subunits deregulated the juxtanuclear positioning of lysosomes and led to an increase in SQSTM1/p62 and lipidated LC3, two autophagic markers, indicative of a decreased lysosomal degradation. Interestingly, this study reveals a requirement of lysosomal dispersal in autophagy that is independent of mTORC1 signaling (Jia et al., 2017).

Neurons are susceptible to the accumulation of cellular waste that cannot be diluted through cell division; thus, lysosomal function and positioning is an imperative process for neuronal health (Menzies et al., 2017). Consequently, the role of BORC has recently been studied in the context of neurodegenerative disease. Namely, mutations in the *Borc7* gene (diaskedin) demonstrably led to progressive axonal dystrophy and the impairment of motor functions in animal models (Snouwaert et al., 2018). Further insight into the implication of lysosomal positioning in neurodegenerative disease could lead to novel therapeutic options. Moving in the opposite direction, retrograde movement of lysosomes is assured by a single cytoplasmic dynein which acts in a dynactin-dependent manner (p150-glued, Arp1) and relies on Rab7 and the Rab7-interacting lysosomal protein (RILP) (Burkhardt et al., 1997; Johansson et al., 2007; Progida et al., 2007; Ishikawa, 2012). Interestingly, the snapin subunit of BORC is also important in retrograde movement through dynein, implicating either the anterograde complex itself or snapin as an integral part of a novel complex in retrograde movement (Cai et al., 2010; Pu et al., 2015). Furthermore, the retrograde movement of lysosomes on microtubule motors over long distances in neuronal cells is critically regulated by JIP3 and JIP4, the loss of which leads to the accumulation of Alzheimer’s disease-related amyloid plaques, also linking retrograde movement to neurodegenerative disease (Drerup and Nechiporuk, 2013; Gowrishankar et al., 2021).

### 5.2 Lysosomal Exocytosis

*Lysosomal exocytosis* is a process where lysosomes fuse with the plasma membrane and release their luminal contents extracellularly (Chavez et al., 1996; Coorssen et al., 1996; Rodriguez et al., 1997; Jaiswal et al., 2002). This is important for physiological processes such as degranulation in cytotoxic T lymphocytes, bone resorption by osteoclasts, parasite defense by mast cells and eosinophils, platelet function in coagulation, hydrolase release by spermatozoa during fertilization and membrane repair (Mostov and Werb, 1997; Tulsiani et al., 1998; Logan et al., 2003; Stinchcombe et al., 2004; Ren et al., 2008; Wesolowski and Paumet, 2011). Lysosomal exocytosis was thought to be limited to specialized secretory cells; it is now characterized as a ubiquitous physiological process. Lysosomal exocytosis is a two-step process: lysosomes relocate next to the plasma membrane and fuse with each other after which an increased intracellular concentration of calcium accompanies lysosomal fusion with the plasma membrane (Rodriguez et al., 1997; Appelqvist et al., 2013). ATP amounts are abundant within lysosomes and can be released in the extracellular space during lysosomal exocytosis to participate in intercellular communication (Ralevic and Burnstock, 1998; Zhang et al., 2007). The molecular machinery mediating lysosomal exocytosis includes the vesicle SNARE (v-SNARE) VAMP7, the Ca^2+^ sensor synaptotagmin VII (SYTVII), the target SNAREs (t-SNAREs) SNAP23 and syntaxin 4 as well as several RAB proteins on the lysosomal surface (Settembre et al., 2013a). Aside from lysosomal content secretion, lysosomal exocytosis is important for plasma membrane injury repair where lysosomes reseal the damaged site (Gerasimenko et al., 2001; Reddy et al., 2001). Lysosomes are thought to be used for this function rather than other organelles because of their excess limiting and internal membranes. They possess all necessary proteins needed for membrane fusion and their content elimination is known to be helpful in ridding lysosomes of any build-up of cellular waste (Blott and Griffiths, 2002). Finally, lysosomal exocytosis is transcriptionally regulated by the transcription factor-EB (TFEB), which induces both the docking and fusion of lysosomes with the plasma membrane by regulating the expression of specific genes which increase lysosomal dynamics and cause an intracellular calcium increase mediated by MCOLN1 (Medina et al., 2011). The importance of lysosomal exocytosis has, sometimes controversially, urged the classification of lysosomes into 2 distinct types; the conventional lysosome, which has a catabolic function and, the secretory lysosome, sometimes referred to as lysosome-related organelles such as melanosomes, immune cell related lytic granules and the late endosomal major histocompatibility complex class II compartment (Raposo and Marks, 2007; Marks et al., 2013). Although very similar in terms of acidity and associated proteins, they have cargo specific properties like perforins and granzymes; furthermore, their biogenesis and secretion remain elusive. Genetic defects in secretory lysosomes have been demonstrated to be involved rare diseases such as Chediak-Higashi disease and Hermansky-Pudlak syndrome (Huizing et al., 2008).

## 6 Lysosome Biogenesis

Another breakthrough in lysosome research was achieved with the discovery of the Coordinated Lysosomal Enhancement And Regulation (CLEAR) regulatory network (Sardiello et al., 2009). The CLEAR network was discovered using co-expression and promoter analyses which led to the identification of a palindromic sequence located at 200bp from the transcription start site, named the CLEAR-consensus sequence. In humans, the microphthalmia-transcription factor E (MiT/TFE) subfamily of bHLH factors is composed of four members; MITF, TFE3, TFEB and TFEC and they bind the CLEAR consensus sequence (Steingrimsson et al., 2004). TFEB and TFE3 are expressed ubiquitously whereas MiTF and TFEC are expressed in specialized cells (melanocytes, osteoclasts, mast cells, macrophages, NK cells, B cells and the heart) and in cells of myeloid origin, respectively. All four members homo- or heterodimerize to activate transcription (Rehli et al., 1999; Massari and Murre, 2000; Meadows et al., 2007; Martina et al., 2014). TFEB positively regulates the expression of lysosomal genes through its subcellular localization which is controlled by mTORC1 phosphorylation at the lysosome (Pena-Llopis et al., 2011). TFEB localizes to both endo-lysosomes and terminal lysosomes (Bright et al., 2016). Under normal nutrient conditions, phosphorylated TFEB (Ser142 and Ser211) is retained in the cytoplasm by binding to the 14-3-3 protein, whereas starvation and mTORC1 inhibition causes its dephosphorylation and its nuclear translocation (Martina et al., 2012; Roczniak-Ferguson et al., 2012; Settembre et al., 2012). Thus, information on lysosomal AA content is relayed to the nucleus via TFEB (Settembre et al., 2012). As with mTORC1, TFEB is also recruited to the lysosome by RagC/D and this recruitment is dependent on the GTPase activating protein for RagC/D, Folliculin (FLCN) (Martina and Puertollano, 2013; Petit et al., 2013). Non-canonically, mTORC1’s inhibitory phosphorylation of TFEB was demonstrated to also be dependent on the Tuberous Sclerosis Complex (TSC) 1 and 2 upstream of mTORC1 in pathogenic TSC models (Alesi et al., 2021). The pathogenic phosphorylation of TFEB was also demonstrated to be RagC dependent. As the inhibition of TSC1 or TSC2 leads to the downstream activation of mTORC1, we would suppose a concomitant phosphorylation and inactivation of TFEB; however, this is not the case. Rather, TFEB remains unphosphorylated, assumes a chiefly nuclear localization, and is accompanied by an increase in lysosome gene expression as well as the proliferation of malignant cells. Interestingly, TSC also shares some clinical phenotype with the Birt-Hogg-Dube (BHD) cancer syndrome which is caused by germline mutations in *FLCN* and which was demonstrated to be TFEB-dependent, reinforcing the importance of RagC in the regulation of TFEB (Schmidt and Linehan, 2018; Napolitano et al., 2020). Further molecular insight in this mechanism is however undoubtfully warranted.

Interestingly, lysosomal Rag GTPases and Tfe3 also plays a critical role in embryonic stem cell differentiation and disease (Villegas et al., 2019). Mechanistically, Ragulator recruits RagC/D to the lysosome where it interacts directly with Ttfe3 to promote its phosphorylation and sequestration in the cytosol which licenses the downregulation of metabolic transcripts and upregulation of developmental transcripts. Interestingly, in contrast to somatic cells, Rag A/B is dispensable to the activation of the Rag GTPases heterodimer complex, and the phosphorylation of Ttfe3 (Saxton and Sabatini, 2017). Functionally, the impairment in the cytosolic retention of Tfe3 which leads to its nuclear localization impedes exit from self-renewal. Interestingly, gain-of-function mutations in the Tfe3 domain, which regulates its cellular localization, has been found in renal cell carcinoma and developmental disorders (Grabiner et al., 2014; Kauffman et al., 2014; Villegas et al., 2019).

In addition, a phosphatase siRNA library revealed new phosphatases responsible for the dephosphorylation of TFEB.(Medina et al., 2015) This study identified a calcium-dependent serine-threonine phosphatase, *calcineurin,* that binds and dephosphorylates TFEB. Moreover, promoter, transcriptome and ChIP-seq analyses performed in HeLa cells identified 471 TFEB direct targets and revealed that the function of the TFEB/CLEAR network was not limited to lysosomal function but also impacted crucial cellular processes of catabolism such as autophagy, exocytosis, endocytosis and immune response (Palmieri et al., 2011; Settembre et al., 2013b).

A fascinating mechanism of lysosomal regeneration was first discovered by Dr. M.J. Lenardo’s research team called *Autophagic Lysosomal Reformation* (ALR) (Yu et al., 2010). By investigating autophagy termination, L.Yu *et al.* discovered this lysosomal mechanism where tubules and vesicles emanate from autolysosomes (AL) in a microtubule-dependent manner and mature into newly functional lysosomes. The tubules detach from the autolysosome through vesiculation and form globular compartments named proto-lysosomes. This elucidated a new model describing autophagy as a continuing circular signaling dependent on mTOR reactivation rather than the classic linear depiction beginning with phagophore formation and ending with lysosome degradation. During prolonged starvation conditions, AL degradation of macromolecules and the subsequent release of intracellular substituents triggers the reactivation of mTOR and the inhibition of autophagy. This negative feedback signal that downregulates autophagy and triggers ALR is dependent on mTOR seen as rapamycin or genetic knock down of mTOR inhibits this process, leaving giant AL. The newly forming proto-lysosomal tubules are positive for lysosomal membrane markers like LAMP-1 but are not acidic and lack enzymes such as cathepsin D, which remain in the vacuolar body of AL. Another study conducted in *Drosophila* showed that the sugar transporter activity of Spinster was required to restore nutrient levels and reactivation of mTOR in this context (Rong et al., 2011). Furthermore, fibroblasts from Lysosomal Storage disease (LSD) patients showed impaired mTOR reactivation and an absence of ALR (Yu et al., 2010). In this cycle of lysosome consumption and reformation, ALR permits the cell to utilize the lysosomal membrane and associated proteins for continued autophagy activation when few resources are available. These studies show that the identified feedback mechanism plays an important role in preventing autophagic cell death by prolonged autophagy and seem to play a crucial role in the pathophysiology of LSDs. The reformation tubules were shown to require the dissociation of Rab7 and the recruitment of clathrin (Rong et al., 2012). The conversion of phosphatidylinositol-4-phosphate(PI(4)P) into phosphatidylinositol-4,5-biphosphate (PI(4,5)P(2)) was required for the recruitment of the clathrin adaptor AP2 that consequently mediated the association of clathrin. Also, fission of proto-lysosomes from the reformation tubules required the recruitment of the GTPase DYNAMIN2 since its depletion prevented fission events (Schulze et al., 2013). PI(4,5)P(2), AP2, DYNAMIN2 and clathrin molecules are localized at the plasma membrane and are well-known for their role in clathrin-mediated endocytosis, thus, it is highly intriguing that this machinery would regulate new, uncharacterized clathrin-mediated sorting events dependent on cellular nutritional status. Contrastingly to clathrin-mediated endocytosis, AP2 recruitment to AL does not require transmembrane proteins but only PI(4,5)P(2) (Schulze et al., 2013) New studies will be needed to answer some key concepts as to how proto-lysosomes acquire their functional set of lysosomal enzymes, whether the mannose-phosphate receptors are required or if undiscovered lysosomal enzyme transport routes are involved. Furthermore, the PAS complex, shown to maintain the functionality of endocytic compartments, calcium channels, and fusion/fission events, has also been shown to initiate terminal lysosome reformation from EL. Indeed, the inhibition of PIKfyve leads to the formation of enlarged EL through homotypic fusion and heterotypic fusion with terminal lysosomes; however, no fission events can be observed leading to the depletion of terminal lysosomes (Bissig et al., 2017). Deeper mechanistic understanding of this process will shed further light on this fascinating process.

## 7 Lysosomal Dysfunctions, Related Diseases, and Therapeutics

### 7.1 Cancer

A predictable and unfortunate outcome of cancer therapeutics is the development of tumor resistance. However, the comprehension of tumor heterogeneity and the molecular complexity of tumor cells within their microenvironment has improved exponentially with high-throughput techniques, bioinformatics, and systems biology. This led to the identification of new targets that are directly implicated in anti-cancer drug resistance (Piao and Amaravadi, 2016). Targeting lysosomes in cancer seemed both promising and innovative because of their roles in homeostatic pathways, autophagy and endocytosis. Together in cancer, these pathways contribute to cell death escape pathways, evading immune surveillance and to metabolism deregulation. Targeting lysosomes is a promising approach as it triggers apoptotic and lysosomal cell death pathways and inhibits the cytoprotective role that autophagy brings to tumor progression (Hanahan and Weinberg, 2011). In fact, lysosomes undergo a series of molecular and functional changes during malignant transformation which leads to tumor aggression, angiogenesis and metastases (Mohamed and Sloane, 2006). To fulfill the excessive needs of cancer cells in the tumor microenvironment, lysosomes become hyperactive. Cancer cells highly depend on lysosomes: they increase lysosomal exocytosis benefitting tumor invasiveness and resistance, they ingest adhesion molecules and large amounts of extracellular matrix, repair membranes, and upregulate their cellular movement and routes (Kallunki et al., 2013; Xu and Ren, 2015; Hamalisto and Jaattela, 2016). Accordingly, striking lysosomal changes include increased lysosome volume, biogenesis and hydrolase activity, and changes in cellular distribution and lysosomal membrane composition (Nishimura et al., 1998; Kroemer and Jaattela, 2005; Gocheva et al., 2006). During malignant transformation, the peripheral population of lysosomes increases and is associated with an increased activity of luminal proteases such as cathepsins B and L (Hamalisto and Jaattela, 2016). This characteristic has led cancer researchers to try and develop novel cancer therapies that could selectively target the particularities of this lysosome population seen as it has been linked to cancer cell migration and invasion. Despite these tumor-promoting aspects, overactive lysosomes exhibit vulnerable and weaker lysosomal membranes that can be exploited and selectively targeted to induce cell death. This increased lysosome sensitivity in tumors can be targeted through Lysosomal Membrane Permeabilization (LMP) causing the release of lysosomal contents in the cytosol (*i.e.* cathepsins) and leading to lysosomal death pathways. The development of new, precise, and sensitive methods for detecting LMP has helped uncover precise mechanisms of cell death triggered by this phenomenon (Aits and Jaattela, 2013; Aits et al., 2015a; Aits et al., 2015b; Jaattela and Nylandsted, 2015). LMP is an attractive method that could be utilized to selectively target metastatic cancer cells whose lysosomes are more vulnerable.

Tumor microenvironment characteristics include hypoxia and acidity and together, they contribute to cancer progression, metastasis and significantly reduce the sensitivity of tumors to chemotherapeutic agents (Fais et al., 2007). In metastatic cancers, V-ATPase pumps are often overexpressed and localize at the plasma membrane. They alter the tumor microenvironment by proton extrusion into the extracellular medium causing extracellular acidification and contributing to the maintenance of an aberrant pH gradient between the mostly alkaline cytosol and the acidic extracellular environment (Dettmer et al., 2006; Fais et al., 2007). This leads to the secretion and activation of several proteases and promotes the degradation and remodeling of the extracellular matrix which drives cancer metastasis (Martinez-Zaguilan et al., 1996; Rofstad et al., 2006). Accordingly, V-ATPase inhibitors were shown to decrease the acidity of tumors and allowed a reduction of tumor metastases (Luciani et al., 2004; Fais et al., 2007). However, weak-base anticancer drugs often accumulate in lysosomes via cation trapping (*i.e.* Sunitinib), thus deviating them from their protein targets within the cells and limiting their effectiveness (Jansen et al., 1999; Gotink et al., 2011; Zhitomirsky and Assaraf, 2015). By traversing freely across phospholipid membranes, these drugs get protonated when exposed to the acidic pH of the lysosomal lumen and are unable to exit (Kaufmann and Krise, 2007; Adar et al., 2012; Zhitomirsky and Assaraf, 2016). In fact, the number of drug-trapped lysosomes per cell correlates with the extent of cellular resistance to these drugs (Zhitomirsky and Assaraf, 2017). Studies by B. Zhitomirsky and Y.G Assaraf show that anticancer drug accumulation in lysosomes triggers their fusion with the plasma membrane and the secretion of their contents (Zhitomirsky and Assaraf, 2017). Indeed, research has linked invasiveness, angiogenesis and tumor progression to extracellular cathepsins (Palermo and Joyce, 2008). These findings suggest that lysosomal exocytosis of chemotherapeutic-loaded lysosomes is a crucial aspect of lysosome-mediated cancer multidrug resistance and could be utilized as new targeted therapies. Although the accumulation of anticancer drugs in lysosomes is mostly due to ion-trapping, some studies have highlighted the active transport of such drugs into the lysosome by ABC transporter P-glycoprotein (Yamagishi et al., 2013). Thus, therapeutically targeting this receptor in combinatory treatment could potentially surmount resistance (Hraběta et al., 2020). However, P-glycoprotein inhibitors have failed in preclinical and clinical studies owing to their toxicity. Instead, repurposing FDA approved drugs with P-glycoprotein inhibitory properties might prove more useful (Lai et al., 2020). Furthermore, recent lines of evidence show that the mechanism by which some cancer cells become resistant to radiation is associated with lysosomal exocytosis, which is enhanced by the activity of Arl8b (Wu et al., 2020). High levels of Arl8b and BORC subunit genes is indeed correlated with a poor prognosis in breast cancer, and knockdown of these genes decreases the survival and invasiveness of cancer cells. These findings point to this pathway as a potential anti-cancer therapeutic avenue.

Targeting lysosomal functions in cancer can also be used to block autophagy and improve cancer therapy effectiveness. Autophagy serves as a survival pathway in cancer, especially in advanced stages and so, blocking its flux could re-sensitize tumors in advanced malignancies. While autophagy can limit tumorigenesis by eliminating damaged proteins and alleviating oxidative stress and genomic instability, it enhances the survival of cancer cells in low-oxygen and low nutrient conditions and engenders therapeutic resistance to cancer therapies in later stages of tumor progression (Mathew et al., 2007a; Xiaohong Ma et al., 2016). In advanced tumors, suppression of autophagy renders cells unable to withstand starvation and results in cell death in response to metabolic stress (Mathew et al., 2007b). However, targeting autophagy remains a challenge because this dynamic process is intimately linked with pathways such as endocytosis. The autophagic process is cell-context dependent and there are varying canonical and non-canonical pathways that, mechanistically, remain to be fully established. Currently, there are five major types of agents used to target lysosomes: chloroquine derivatives (CQ), v-ATPase inhibitors, acid sphingomyelinase modulators (ASM), cathepsin and HSP70 inhibitors (Smith and Schuchman, 2008; Lankelma et al., 2010; Granato et al., 2013; Maynadier et al., 2013; Duong et al., 2014; Kos et al., 2014; Tsai et al., 2014; Piao and Amaravadi, 2016). While most of them are utilized in fundamental research, chloroquine derivatives have shown potential in cancer therapy and have reached clinical trial; however, a recurrent problem with CQ derivatives is that high doses were required for an effective autophagy blockage in human tissues, which produced dose-limiting toxicities (Rebecca and Amaravadi, 2016). PIKfyve is also being considered as a potential target against autophagy-dependent cancers and new molecules have been identified *in vitro* for this purpose, namely, the WX8 series of potent inhibitors (Sharma et al., 2019). Furthermore, combinatory treatments of novel PIKfyve inhibitor, ESK981, with immune checkpoint blockade in castration-resistant prostate efficiently decreased tumor growth in preclinical models (Qiao et al., 2021). The proof of concept demonstrated in this study will be further investigated in phase II clinical trials alone or in combination treatments with the immune checkpoint blockade therapy, nivolumab. This, however, will not be the first use of a PIKfyve inhibitor in clinical trials for the treatment of various diseases, including cancer (Sands et al., 2010; Krausz et al., 2012; Cai et al., 2013; Nelson et al., 2017). The PIKfyve inhibitor, Apilimod, initially proved hopeful against B-cell malignancies; however, this approach still requires progress in terms of pharmacokinetics, cell death mechanism and the potential use of combinatory treatments (Gayle et al., 2017; Ikonomov et al., 2019).

### 7.2 Lysosomal Storage Diseases

Lysosomal Storage Disorders started being characterized in the 19th century by leading physicians whose surnames were used to name the disorders *i.e.* Warren Tay, Bernard Sachs, Phillipe Gaucher, Johanness Fabry, Albert Niemann, Ludwig Pick and Joannes Pompe (Tay, 1881; Gaucher, 1882; Sachs, 1887; Fabry, 1898; Niemann, 1914; Pick, 1926; Pompe, 1932).Today, close to 60 LSDs are identified and are caused by mutations in genes implicated in lysosomal integrity such as lysosomal hydrolases and lysosomal membrane proteins (Coutinho and Alves, 2016). LSDs are multisystemic and affect a plethora of organs including the skeletal system, muscles, liver and spleen, heart, lungs and the central nervous system, the latter being the leading cause of most pediatric neurodegenerative diseases (Coutinho and Alves, 2016). Biochemically, LSDs are characterized by the accumulation of un- or partially digested molecules inside lysosomes. Lysosomes are implicated in a multitude of signaling pathways comprising specialized functions and for this reason, a compromised lysosomal catabolism will inevitably impede cellular functions such as: 1) defects in glycosaminoglycan, lipid, or protein degradation; 2) transport across the lysosomal membrane or 3) vesicle trafficking. Consequently, a novel classification of LSDs is now emerging including groups of disorders that are due to defects in non-enzymatic lysosomal protein such as transmembrane protein (transporters and structural proteins), lysosomal enzyme protection, post-translational processing of lysosomal enzymes, trafficking of lysosomal enzymes and polypeptide degradation. Even though LSDs are caused by a single defective protein, accumulation of non-degraded material will block later stages of autophagy (Reggiori and Klumperman, 2016). Accordingly, diverse studies are highlighting the close interconnection between LSDs and an impaired autophagic flux (Lieberman et al., 2012). Defects in autophagy is now a common feature of LSDs. In many cases, their pathology often involves neurodegeneration caused by impaired autophagy and accumulation of cytoplasmic aggregates leading to neuronal cell death (Komatsu et al., 2006; Pastores and Maegawa, 2013). Thus, the current LSD classification is based on the nature of the accumulated substrate(s) and many enzyme deficiencies are now associated to some LSDs: β -glucocerebrosidase (GCase) underlying Gaucher disease, α -galactosidase triggering Fabry disease, sphingomyelinase causing Niemann-Pick disease and α -L-Iduronidase or iduronate sulfatase underlying Hurler and Hunter syndromes (Brady et al., 1966; Kanfer et al., 1966; Fratantoni et al., 1968).

Enzyme replacement therapies have been approved and are marketed for the treatment of a few LSD, namely Gaucher disease (type 1), Fabry disease, and Pompe disease, amongst others (Platt, 2018). Indeed, this strategy was initially pioneered over 4 decades ago to treat patients suffering from Gaucher disease (Brady, 2006). Functionally, the treatment consists in the administration of a functional enzyme via mannose or mannose-6-phosphate receptors for the delivery of the enzyme to the lysosome where it can degrade its substrate (Platt, 2018). Although considered an effective treatment, a clear limitation remains the inability of recombinant enzymes to cross the blood-brain barrier, rendering the treatment inefficacious for central nervous system manifestations (Safary et al., 2018). The more invasive delivery of enzymes by way of intracerebroventricular and intrathecal administration is thus a potential alternative delivery route (Desnick and Schuchman, 2012; Platt, 2018). Furthermore, this strategy relies on the delivery of functional enzymes via membrane receptors that may not be heavily expressed, as seen in skeletal muscles; hence, high doses are needed for the treatment of Pompe disease, for example (Platt, 2018). Modifying the enzyme for greater receptor accessibility could potentially aid in overcoming this hurdle (Platt, 2018). Furthermore, substrate reduction therapy presents an alternative therapeutic avenue. These therapies consist of small molecules that can reduce substrate accumulation by inhibiting enzymes involved in substrate synthesis. In fact, some therapies have been approved, such as miglustat and eliglustat, and others are presently undergoing clinical trials (Lucerastat, ibiglustat and genistein) (Radin, 1996; Hughes et al., 2017; Guérard et al., 2018; Pineda et al., 2018). Stabilizing mutated enzymes using substrate mimics has also proved potentially beneficial, such as seen with marketed migalastat (Fabry disease), and other drugs in clinical trials; pyrimethamine (Sandhoff disease, phase I), afegostat (Gaucher disease, failed phase II), and ambroxol (Gaucher disease, suspended phase I/II) (Steet et al., 2006; Hughes et al., 2017; Magalhaes et al., 2018; Bonam et al., 2019). The screening of small molecules able to ameliorate the functionality of mutated proteins has highlighted another promising approach. In a screen, NCGC607 was able to reduce lysosome substrate accumulation in stem cells derived from patients diagnosed with Gaucher disease; however, further testing is needed to test its effectiveness in LSD patient directly (Aflaki et al., 2016; Platt, 2018). Gene therapy for the treatment of LSD has also started to garner attention, with preclinical and clinical trial underway (Sevin and Deiva, 2021). Altogether, more clinical trials are needed to determine the robustness of these approaches.

LSDs also significantly contribute to cardiovascular disorders with patients presenting severe phenotypes such as hypertrophic and dilated cardiomyopathy, coronary artery disorders, and valvular defects (Chi et al., 2020). Cardiac involvement is most often found in glycogen storage diseases such as Pompe and Danon diseases (Nair et al., 2019). In fact, Pompe and Danon diseases as well as Mucopolysaccharidosis (MPS, a group of storage diseases caused by loss of function mutations of lysosomal enzymes responsible for the degradation of glycosaminoglycans) are all types of LSDs that can have direct repercussions on cardiac function (Braunlin et al., 2011; Chi et al., 2020). In Pompe disease, several cardiac complications have been observed such as enlargement of the heart, thickening of the left ventricular free wall and the papillary muscles, severe wall thickening associated with obstruction of the left and right ventricular outflow, cardiac dilatation, and fibroelastic thickening of the endocardium (Wenger et al., 1986; Nair et al., 2019) In Danon disease, the cardiac manifestations are generally more severe in men and include left ventricular hypertrophy and Wolf Parkinson’s White syndrome, a condition of electrical abnormality in the heart that can cause tachycardia (Stokes et al., 2016). Finally, individuals with MPS suffer from coronary artery narrowing and occlusion and generalized cardiomegaly and calcification of the mitral valve ring (Raben et al., 2002; Nair et al., 2019).

### 7.3 Neurodegenerative Diseases

Several neurodegenerative disorders, namely Alzheimer disease (AD), Parkinson disease (PD), and Huntington disease (HD) are the result of inadequate disposal of neurotoxic proteins by the lysosomal pathway (Boland et al., 2018). For instance, in AD, the amyloid precursor protein (APP) is cleaved into amyloid-β peptide (Aβ) fragments, the accumulation of which leads to alterations in the lysosomal membrane, ultimately resulting in neuronal cell death (Zhang et al., 2009). Patients with inherited forms of AD have also presented with mutations in presenilin genes (PSEN1 and PSEN2). This results in the loss of lysosome acidification by the misrouted V0A1 subunit of the v-ATPase, having a deleterious effect on lysosomal functioning (Lee et al., 2010; Lauritzen et al., 2019). The accumulation of phosphorylated tau in neurofibrillary tangles has also been considered endemic in AD (Hampel et al., 2010). Cathepsin D has notably been shown to be involved in the cleavage of tau proteins into tangle fragments. Indeed, the inhibition of cathepsin D in the brain of patients with AD was shown to diminished its hyperphosphorylation (Bi et al., 2000). TFEB has also been shown to be selectively lost in AD patients (Wang et al., 2016). Increasing TFEB activity might prevent neuronal death in this case (Torra et al., 2018). Several genes directly or indirectly linked to endocytic and lysosomal physiology have also been documented in PD, for instance, mutations in β -GCase (the causative gene of Gaucher disease) and α-synuclein (Zhang et al., 2009; Dehay et al., 2013; Aflaki et al., 2017). Mutated α-synuclein is unable to be degraded via its regular route (CMA pathway) and eventually forms macro-aggregates that further impede the degradation of other cargo by binding and inhibiting LAMP2A. Fibrillar α-synuclein accumulates and form neurotoxic protein inclusions named Lewis bodies. HD is a rare autosomal-dominant neurodegenerative disease caused by the *HTT* gene. The mutated gene disrupts post-Golgi trafficking leading to the misrouting of optineurin/RAB8 complex, also negatively affecting lysosome physiology (del Toro et al., 2009). Altogether, there is increasing evidence for the role of lysosomes in neurodegenerative disease; therefore, these organelles may present a novel target for their treatment.

## 8 Discussion

The growing interest in lysosome research has led to some ground-breaking discoveries by passionate and talented researchers in the field. Elegant mechanistic studies have now characterized these acidic organelles as being much more complex than originally thought. In addition to their fascinating mechanisms of biogenesis and reformation, they are the key to cellular nutrient sensing, communicators of energy necessities. Most importantly, lysosomes are linked to essential pathways such as autophagy, endocytosis as well as vesicle trafficking and recycling routes placing them at the center of highly dynamic processes. Open questions regarding lysosome ionic channels’ biophysical properties and functionality still need investigating, as well as the regulation of the PIKfyve complex and retrograde movement of the lysosome. As defects in this pathway have been linked to rare hereditary diseases as well as neurodegeneration and cancer, deep insight into the molecular mechanism of lysosome physiology could help in the molecular characterization of disease as well as guide the development of new therapeutic strategies. In the context of cancer pathophysiology, cancer cells become highly dependent on lysosomal functions making it possible to exploit this characteristic by selectively targeting weakened lysosomes, an approach that has proven highly promising. However, to fully exploit oncogenic lysosomal changes, we need to continue to uncover and understand non-canonical compensation mechanisms utilized by transformed cells. Novel tools are desperately needed to better understand the complex symphony existing between these dynamic pathways, where their interdependence is very much cancer-type and stage-dependent. Elucidating mechanistic insights in these processes in several cancer models will undeniably lead to the identification of effective single- and combinatorial drug therapies for new targetable effectors in cancer.
